# Social Well-Being and Chronic Disease Condition among Older Adults

**DOI:** 10.1093/geroni/igaa057.3287

**Published:** 2020-12-16

**Authors:** Jungkyung Min, Idethia Harvey, Yuchen Yeh

**Affiliations:** Texas A&M University, College Station, Texas, United States

## Abstract

Aging is characterized by the decline in physical health, functional status, and loss of social roles and relationships that can challenge the quality of life. A protective factor that moderates the impact of these phenomena is psychological (e.g., social) well-being. Despite the high prevalence of chronic conditions among older adults, research exploring the relationship between social well-being and chronic disease is sparse. The study aims were to investigate the relationship between social well-being among older adults (N = 1,251, R = 65 – 92 years) who participated in the National Survey of Mid-life in the United States (MIDUS 3). This study used variables for the MIDUS 3 study to test a structural equation model to examine the hypothesized relationships between social well-being, chronic conditions, life satisfaction, self-esteem, active coping, optimism, and religious coping. The findings indicate that perceived control, self-esteem, active coping, optimism, and religious coping were statistically significant for the participants’ social well-being (β =.29, p <.001, β =.16, p<.001, β =.08, p<.05, β =.35, p<.001, and β =.07, p<.05, respectively). However, life satisfaction was not significantly associate with social well-being (β =.04, p >.05). For individuals’ diagnosed with more than one chronic condition, perceived control, self-esteem, and optimism statistically significant impact their social well-being (β = .33, p < .001, β =.17, p < .001, and β =.33, p < .001, respectively). Findings suggested that multiple chronic conditions influence social well-being. Chronic disease management programs may be useful in increasing social well-being among individuals with multiple chronic conditions.

